# Society of Biology

**Published:** 1879-04

**Authors:** O. C. Oliver


### ARTICLE XIII.

SOCIETY OF BIOLOGY. President, M. Paul Bert. Extracts from the transactions at the meeting of Feb. 8, 1879.

Mons. Mathias Duval, professor of histology in the faculty of medicine, stated that he preferred a mixture of collodion and alcohol for imbedding tissues of which it might be desired to make sections, instead of the "wax and oil" mixture so generally employed, which is opaque and prevents a clear view of the portion of tissue to be cut, while the former mixture is transparent, and has the additional advantage of a certain degree of elasticity very favorable for obtaining thin sections, of which a number of very fine ones were exhibited, chiefly of embryos.

M. Laborde communicated the results of his experiments on the effect of injections of milk into the veins. He found that the injection of 80 cubic centimeters of milk into the veins of a dog weighing 11 or 12 kilos. caused very rapid death, but repeated injections of 25 to 30 grams were easily borne. Almost immediately after the injection, milk globules were found in the general circulation, the larger always appearing last. Little by little the globules disappeared from the circulation, to accumulate in the "splanchnic organs." In those animals who died in from 20 to 40 hours, M. Laborde found ecchymoses in the lungs and mucous membrane of the stomach. In the centers of these hæmorrhagic spots, he found numerous white globules histologically identical with those of milk. Numerous capillary hæmorrhages were also found in different portions of the encephalon. M. Laborde has also experimented upon frogs, in which class of animals the injection of minute quantities of milk was made with an invariably fatal result. The injections were made into the lymph sacs of this latter animal.

The speaker stated that he considered it prudent to abstain from the injection of milk into the vascular system of man, as had been done by some physicians in the treatment of cholera, except in the so-to-speak desperate cases, under which circumstances alone he considered this bit of tentative practice justifiable. The speaker had been able to preserve a dog in whom fractional doses of milk had been injected into the veins, after a venesection, of 200 grams. If the dog should survive, he would be shown at the next meeting. The president stated that he had also experimented upon dogs by injections of milk into the veins, but the results were too inconstant to permit deductions of any practical avail in the treatment of man.

Dr. Brown-Séquard said his experiments upon these classes of animals had led him to doubt very gravely the therapeutic efficacy of this measure in the treatment of disease, and that he considered the practice indefensible, except in hopeless cases.

M. DeSintef gave reasons why intra-vascular injections of milk were to be considered very dangerous. Fresh milk was certainly least so, but even so soon as 40 minutes after having left the udder, there formed around the globules a film of coagulation, thus constituting a supplementary envelope, which, according to his investigations of the emboli caused by the injections, produced the infarctions. According to M. Laborde, the red blood globules assimilated the milk globules, which latter surrounded the former, "fusing," as it were, and constituting an envelope of a peculiar fringed appearance, which rapidly dissolved in ether, while the red globule remained intact. Laborde sacrificed a young dog a short time after an injection of milk into the veins, and, as a check experiment, another of about the same age, in which no injection had been made. In the first instance, the appearances of the red globules were found to be quite characteristic.

M. Ranvier wished to make some formal reserves (?) upon this subject of the assimilation of the milk globules by the red blood globules. He wished to remind histologists that the blood contained fat globules, yet it certainly seemed to him that it would be impossible to dissolve these by ether without affecting the red globules themselves. O. C. OLIVER.

